# PERCEPTIONS OF THE “GOOD LIFE” AMONG NURSING HOME RESIDENTS WITH DEMENTIA, FAMILY, AND STAFF

**DOI:** 10.1093/geroni/igae098.0071

**Published:** 2024-12-31

**Authors:** Jessica Benfer, Gretchen Tucker, John Cagle

**Affiliations:** Meros Center, Milwaukee, Wisconsin, United States; University of Maryland Baltimore, Baltimore, Maryland, United States; University of Maryland Baltimore, Baltimore, Maryland, United States

## Abstract

Providing high quality care to nursing home residents with dementia and enhancing their quality of life is complicated by a variety of noteworthy challenges (e.g., staff turnover, difficult transitions, quarantines). Our objective was to characterize perceptions of the “Good Life” among nursing home residents with dementia, families, and staff from a nationally derived sample of nursing homes. Using Gitlin’s “Good Life” model as a conceptual framework, 46 mixed-method interviews were conducted over the phone among 11 residents with dementia, 19 family members, and 16 staff from seven nursing homes across the United States. For resident interviews, family members or staff were present to help facilitate the interviews. Data were analyzed thematically. Overall, efforts were viewed as positive in nursing homes to support engaging environments for residents with dementia. Residents in our sample had better days when there was an easy-to-follow schedule of daily events and they felt connected with others, including having positive interactions with staff. Family members reported better days when they were reassured that they were doing what is right for their loved one and could balance caretaking with the rest of their lives. Staff were primarily concerned that residents were well cared for and their needs were met. Findings provide insight into several potential avenues to better promote the Good Life among nursing home residents with dementia, their families, and staff.

